# Supplementary material for: Perinatal murine cytomegalovirus infection reshapes the transcriptional profile and functionality of NK cells
Source: Nat Commun. 2023 Oct 12;14:6412. doi: 10.1038/s41467-023-42182-w (PMC10570381; doi:10.1038/s41467-023-42182-w)
Supplement: Supplementary file 1 — Supplementary information [file 41467_2023_42182_MOESM1_ESM.pdf]

Supplementary information

Perinatal murine cytomegalovirus infection reshapes the transcriptional profile and functionality of NK cells

Rožmanić et al.

Table of contents:

Supplementary Figures 1-11

Supplementary Methods

Supplementary References

Supplementary figures:

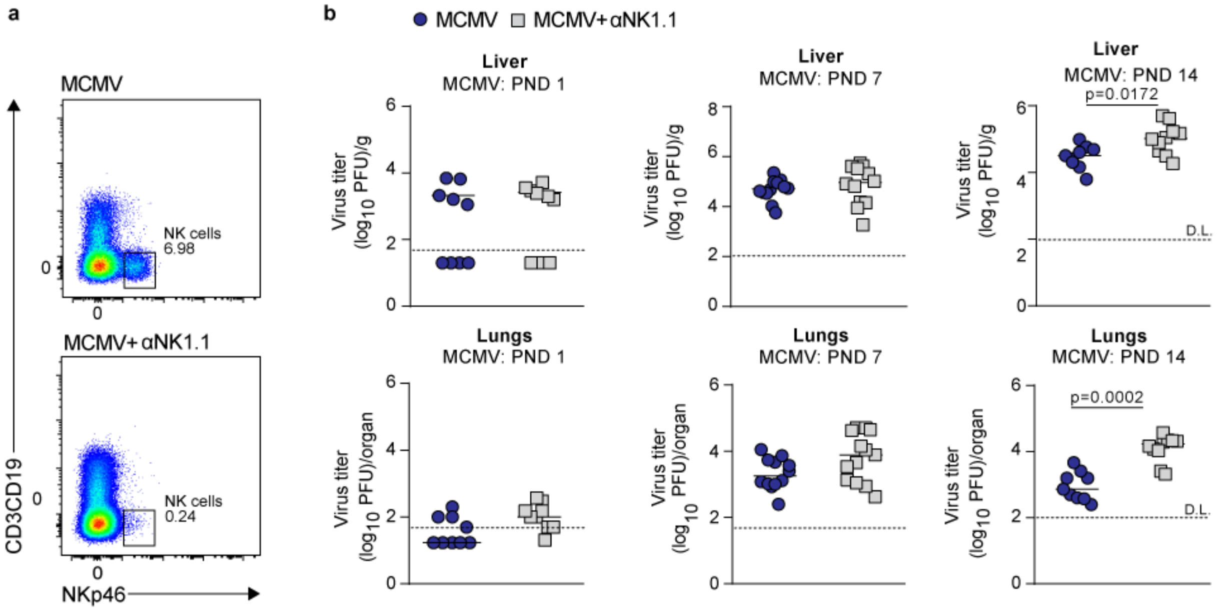

Supplementary Fig. 1: C57BL/6 mice were intraperitoneally infected with MCMV on different postnatal days (PND), as indicated. Viral doses used for infection: 200 PFU for PND 1, 30 000 PFU for PND 7, and 50 000 PFU for PND 14. Indicated groups of mice were depleted of NK cells (αNK1.1). (**a**) Depletion of NK cells in liver was verified using flow cytometry. Representative dot plots are shown, N = 3. (**b**) Virus titers were determined in the liver and lungs four days after infection. (PND 1 n = 9 mice per group; PND 7 n = 12 mice per group; PND 14 n = 9 MCMV, n = 10 MCMV+αNK1.1, Mann-Whitney two-tailed test). D.L., detection limit. Statistically significant differences are indicated (P values). Source data are provided as a Source Data file.

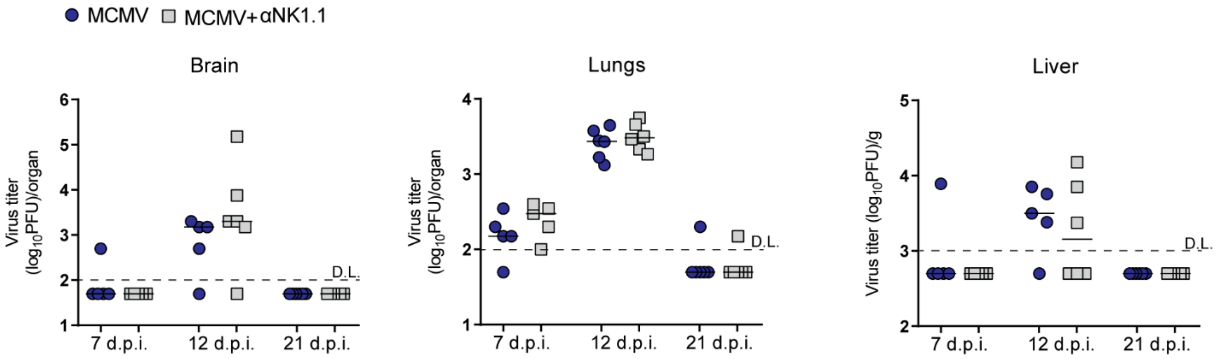

Supplementary Fig. 2: Newborn C57BL/6 mice were intraperitoneally infected with 200 PFU of MCMV 24-36 h after birth. The indicated groups of mice were depleted of NK cells (αNK1.1). At designated time points (days post infection, d.p.i.), virus titers were determined in indicated organs (n = 5 mice per group). D.L., detection limit. Source data are provided as a Source Data file.

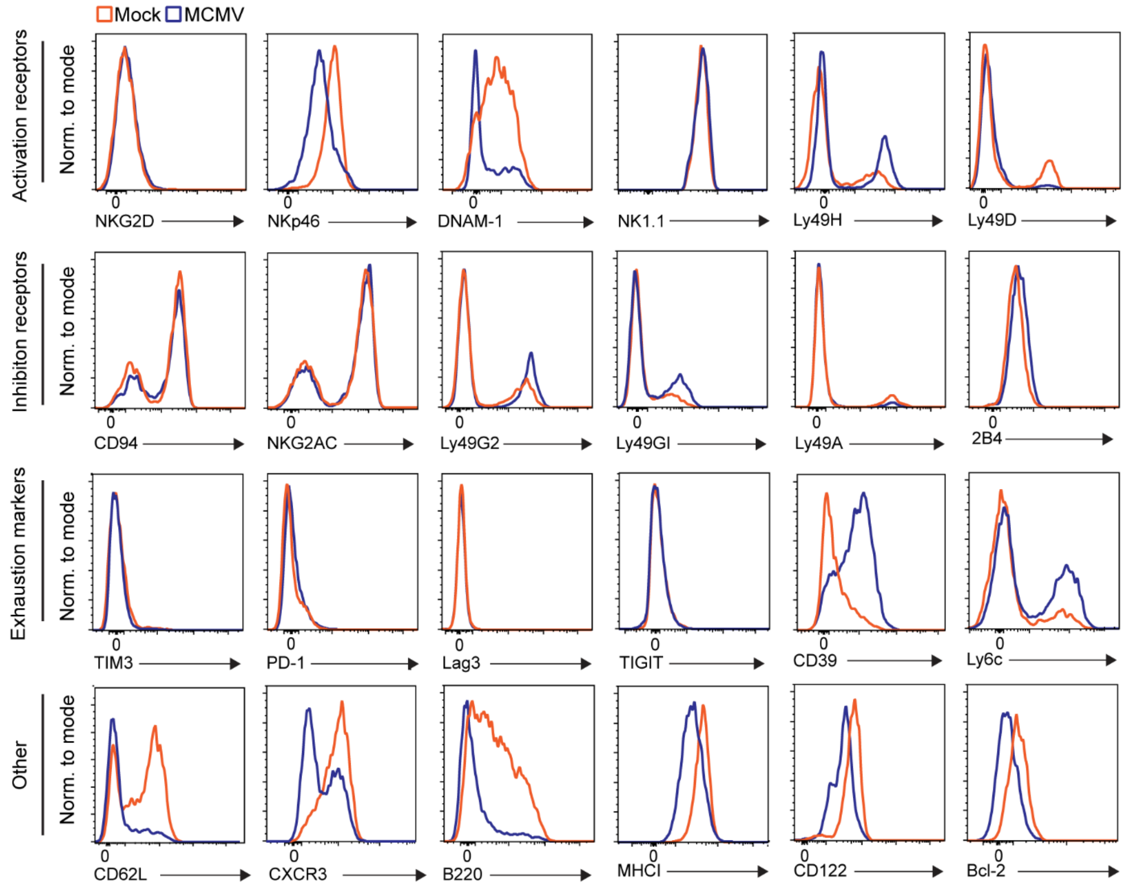

Supplementary Fig. 3: Newborn C57BL/6 mice were intraperitoneally infected with 200 PFU of MCMV or Mock infected 24-36 h after birth. On day 21 p.i. expression of indicated markers was analyzed by flow cytometry. Representative histograms are shown (N = 3).

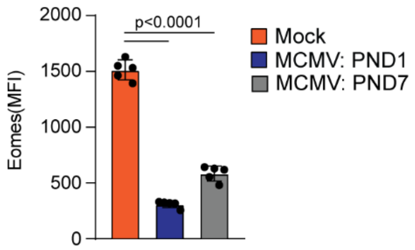

Supplementary Fig. 4: Newborn C57BL/6 mice were infected with 200 PFU of MCMV on postnatal day (PND) 1 or with 400 PFU on PND 7. On day 21 after infection mice were euthanized and spleens harvested. Expression of Eomes on NK cells was analyzed by flow cytometry. Mean values ± SEM are shown (*n* = 5 mice per group, One-way ANOVA test). Statistically significant differences are indicated (P values). Source data are provided as a Source Data file.

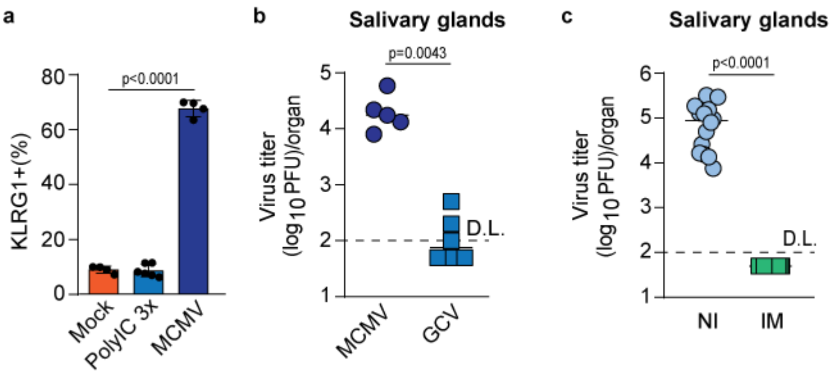

Supplementary Fig. 5: (a) Newborn C57BL/6 mice were infected with 200 PFU MCMV, mock-infected or treated with PolyIC, as indicated. On day 21 p.i. expression of KLRG1 on NK cells was analyzed using flow cytometry. Mean values ± SD are shown (*n* = 5 mice per group, One-way ANOVA test). **(b)** Newborn C57BL/6 mice were infected with 200 PFU of MCMV. Indicated groups were treated with ganciclovir (GCV) every day post infection. On day 10 p.i. mice were euthanized and salivary glands harvested. Titers of individual mice (circles/squares) and median values (horizontal bars) are shown. D.L., detection limit. (MCMV *n* = 5, GCV *n* = 6, Mann-Whitney two-tailed test). **(c)** C57BL/6 female mice were immunized intravenously with $2x10^5$ PFU of Δm157MCMV (immunized, IM) or uninfected (non-immunized, NI), 2 weeks before mating. 2 weeks after infection females were mated with naïve male mice. Newborn mice were intraperitoneally injected with 200 PFU of MCMV 24-36 h after birth and viral titers were determined in salivary glands 21 days p.i. Titers of individual mice (circles/squares) and median values (horizontal bars) are shown. D.L., detection limit. (*n* = 13 mice per group, pooled data of N = 2, Mann-Whitney two-tailed test). Statistically significant differences are indicated (P values). Source data are provided as a Source Data file.

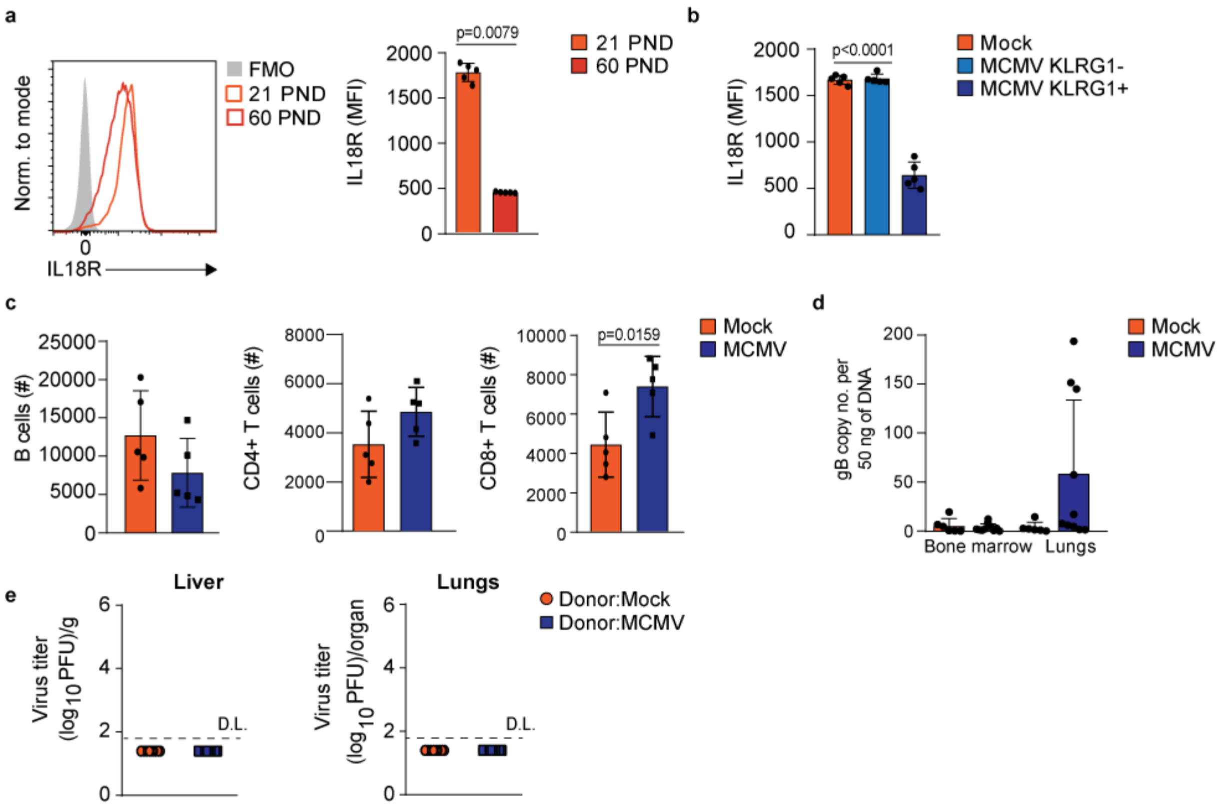

Supplementary Fig. 6: (**a**) Expression of IL18R on splenic NK cells was analyzed in 10 and 60 days old mice; postnatal day, PND. Representative histograms and mean values ± SD are shown (*n* = 5 mice per group, Mann-Whitney two-tailed test). (**b**) Newborn mice were intraperitoneally infected with 200 PFU of MCMV or mock-infected 24-36 h after birth. On day 21 after infection, expression of IL18R was determined by flow cytometry. Quantification of IL18R expressing KLRG1^{+} and KLRG1^{-} NK cells from MCMV-infected mice and NK cells from mock-infected mice is shown (*n* = 5 mice per group, One-way ANOVA test). (**c-e**) Newborn mice were intraperitoneally infected with 200 PFU of MCMV or mock-infected 24-36 h after birth. (**c**) On day 21 post-infection, C56BL/6 mice were euthanized, and bone marrow cells were isolated and transferred into *Rag2*$^{-/-}$*γc*$^{-/-}$ mice. Seven days after adoptive transfer, recipient mice were euthanized, and the number of splenic B cells, CD4+ T cells and CD8+ T cells was determined (*n* = 5 mice per group, Mann-Whitney two-tailed test). (**d**) Number of genomes were determined by qPCR in bone marrow cells and lungs of mock- and MCMV-infected C57BL/6 mice. (**e**) Viral load in the liver and lungs of *Rag2*$^{-/-}$*γc*$^{-/-}$ mice that received bone marrow from Mock- or MCMV-infected mice (*n* = 10 mice per group, pooled data of N = 2). Statistically significant differences are indicated (P values). Source data are provided as a Source Data file.

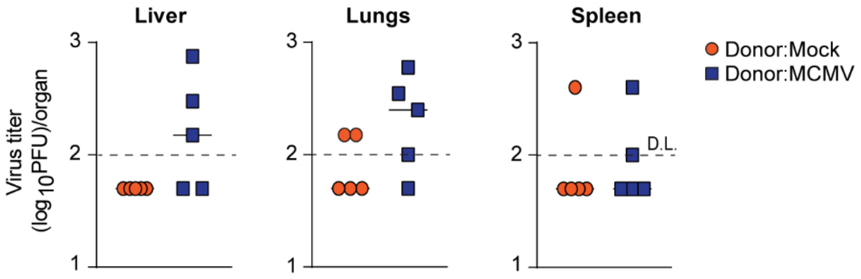

Supplementary Fig. 7: Newborn C57BL/6 mice were infected with 200 PFU of MCMV. On day 21 after infection mice were euthanized and spleens harvested. Splenic NK cells from MCMV or Mock infected mice were enriched and then adoptively transferred into MCMV infected newborn mice 2 days post infection. Viral titer was determined in indicated organs 4 days after transfer. Titers of individual mice (circles/squares) and median values (horizontal bars) are shown. D.L., detection limit (*n* = 5 mice per group). Source data are provided as a Source Data file.

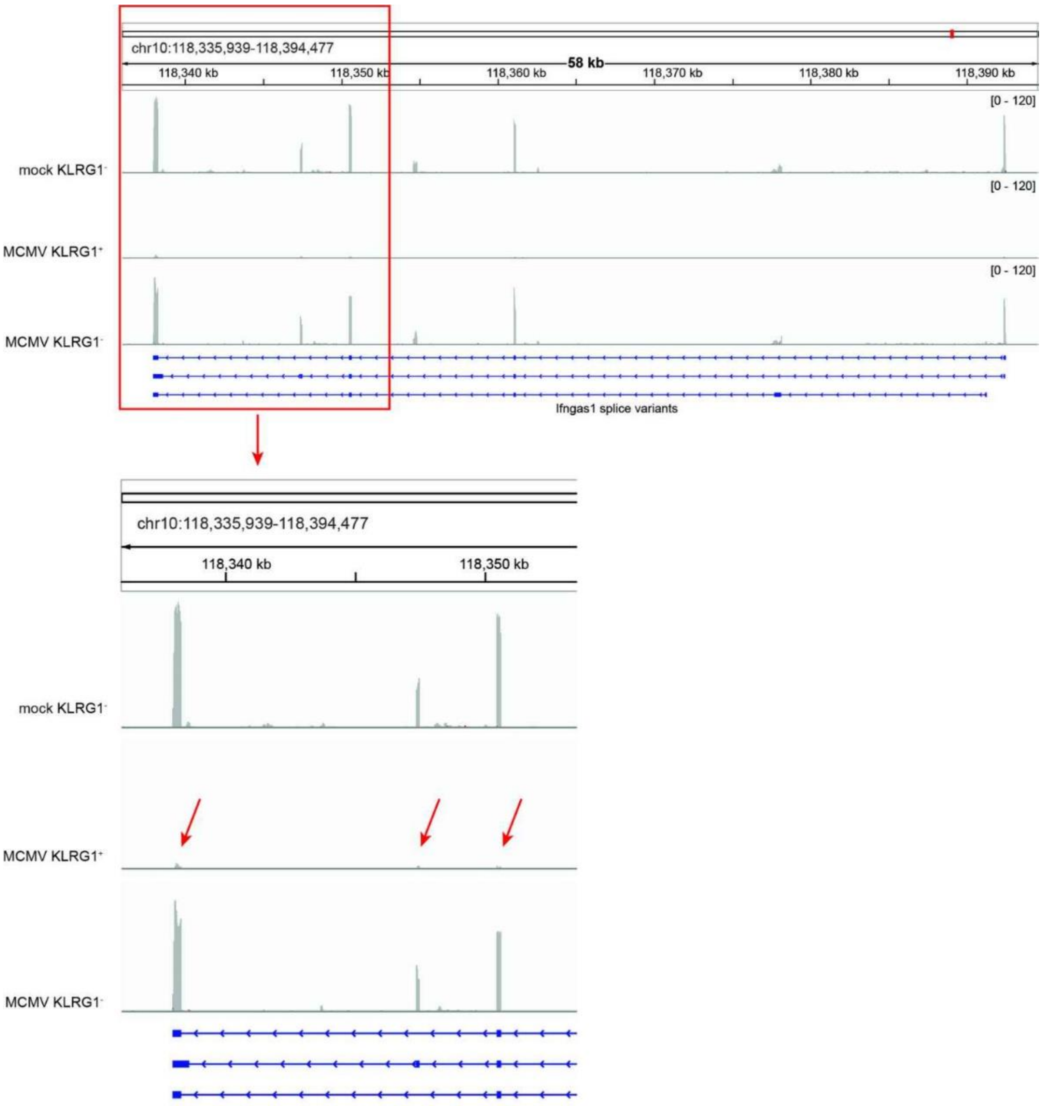

Supplementary Fig. 8: RNA-Seq read coverage at the mouse *Ifng-as1* locus. Reads obtained by RNASeq of KLRG1$^-$ NK cells from mock-infected mice (mock KLRG1$^-$), KLRG1$^+$ cells from MCMV-infected mice (MCMV KLRG1$^+$), and KLRG1$^-$ NK cells from MCMV-infected mice (MCMV KLRG1$^-$) were aligned to the mouse genome sequence as described in the Materials and Methods. Read alignments and coverage depth at the *Ifng-as1* locus were then visualized using Integrative Genomics Viewer v2.16.1 (Robinson, Thorvaldsdottir et al. 2011, Thorvaldsdottir, Robinson et al. 2013). Top-panel shows read coverage depth across the entire *Ifng-as1* gene locus, whereas the bottom panel shows read coverage at its 3'-end.

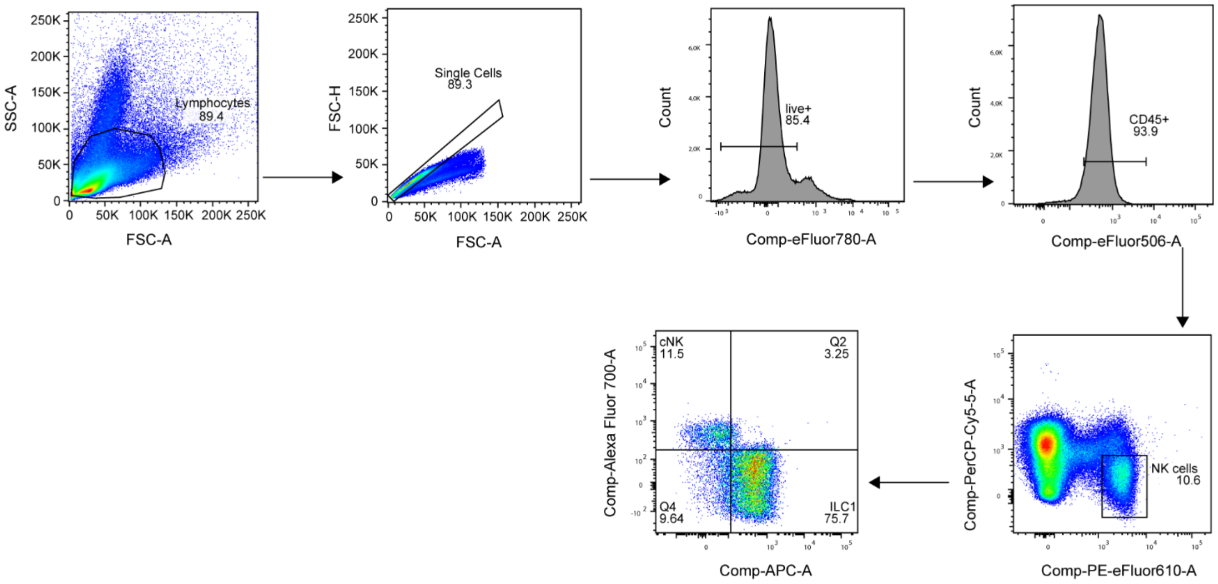

Supplementary Fig. 9: Gating strategies for identification of NK cells (cNK) and ILC1 cells.. Livers were passed through a 70-µm-pore-size nylon cell strainer (BD Falcon, Franklin Lakes, NJ) and washed with 3% RPMI. Cell suspensions were then layered onto two-step discontinuous Percoll gradients (Pharmacia Fine Chemicals, Piscataway, NJ) for density separation. Hepatic leukocytes were collected after centrifugation for 30 min at 900 × g, followed by erythrocyte lysis. Lysis was blocked by adding media, after which cells were centrifuged for 5 min at 300 x *g* and resuspended in fresh media. Before staining of lymphocytes, Fc receptors were blocked using a 2.4G2 antibody. Cells were labeled with fixable viability dye (eFluor780) to exclude dead cells and with anti-CD45.2-eFluor506, -CD3-PerCP-Cy5.5, -CD19-PerCP-Cy5.5, -NK1.1-PE-eFluor610, -CD49a-APC and -CD49b-Alexa Fluor700. NK and ILC1 cells are defined as live CD45.2+CD3-CD19-NK1.1+ cells, NK cells being CD49b+CD49a- and ILC1s being CD49b-CD49a+.

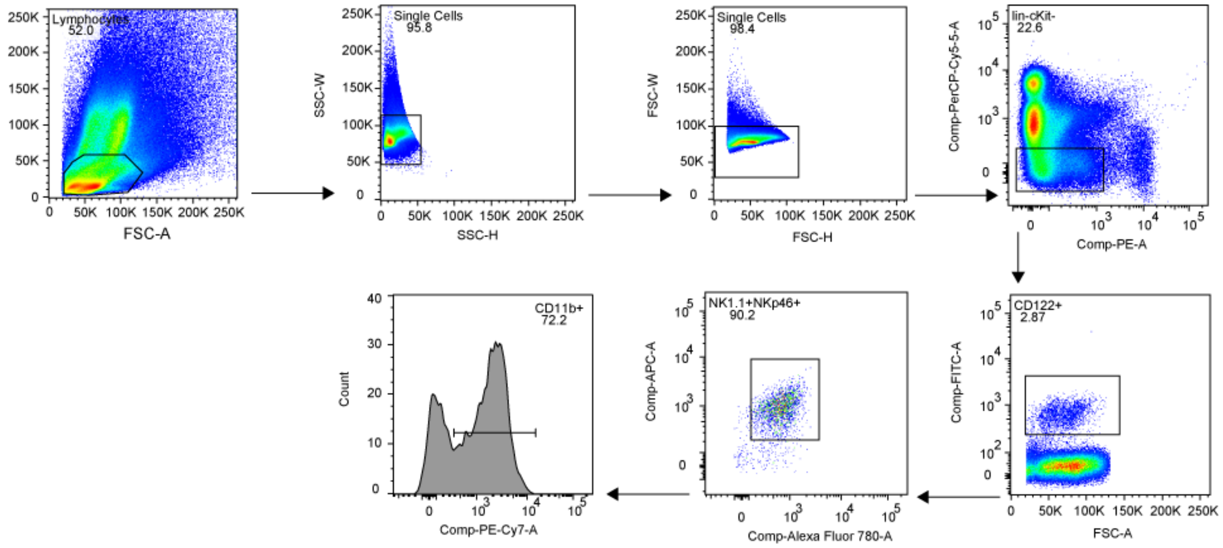

Supplementary Fig. 10: Gating strategies. For bone marrow isolation the femur and tibia were separated by overextending and twisting the knee joint. Any additional muscle or connective tissue attached to the femur was removed and the bones were gently opened at the knee end. The bones were placed in 0.5 mL microcentrifuge tube, previously pierced with a 18G needle at the bottom and nested in a 1.5 ml microcentrifuge tube, knee-down and centrifuged 10 000 x g for 20 sec followed by erythrocyte lysis. Lysis was blocked by adding media, after which cells were centrifuged for 5 min at 300 x g and resuspended in fresh media. Before staining of lymphocytes, Fc receptors were blocked using a 2.4G2 antibody. Cells were labeled with fixable viability dye (eFluor780) to exclude dead cells and with anti-CD3-PerCP-Cy5.5, -CD19-PerCP-Cy5.5, -NK1.1-APC, -NKp46-Alexa Fluor700, -CD11b-PE-Cy7, cKit-PE and -CD122-FITC. NKP are defined as CD122+, cKit-, NK1.1-, NKp46-, CD19-, CD3-, CD11b-, iNK as CD122+, cKit-, NK1.1+, NKp46+, CD19-, CD3-, CD11b- and mNK as CD122+, cKit-, NK1.1-, NKp46-, CD19-, CD3-, CD11b+.

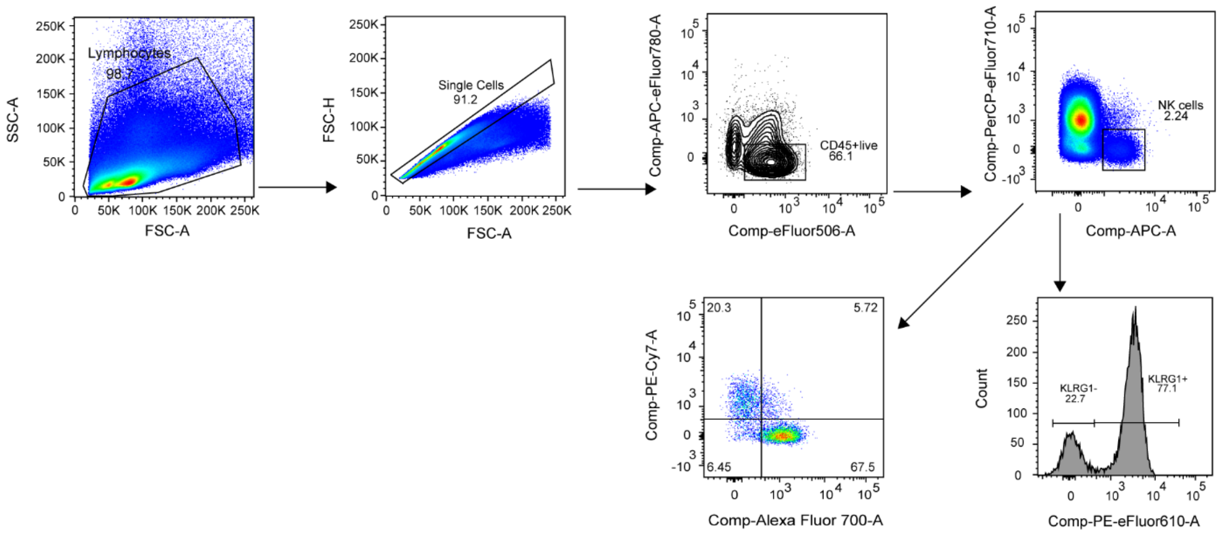

Supplementary Fig. 11: Gating strategies. Spleens were harvested and homogenized, followed by erythrocyte lysis. Lysis was blocked by adding media, after which cells were centrifuged for 5 min at 300 x g and resuspended in fresh media. Before staining of lymphocytes, Fc receptors were blocked using a 2.4G2 antibody. Cells were labeled with fixable viability dye (eFluor780) to exclude dead cells and with anti-CD45.2-eFluor506, -CD3-PerCP-Cy5.5, -CD19-PerCP-Cy5.5, -NK1.1-APC, -CD11b-Alexa Fluor 700, -CD27- PE-Cy7 and -KLRG1- PE-eFluor610. NK cells were defined as live CD45.2+CD3-CD19-NK1.1+ cells.

Supplementary Methods:

RNA-Seq

RNA sample preparation, quality control and sequencing. To determine changes in NK cells in the postnatal period, splenocytes were isolated from spleens of naive mice at postnatal days 7, 14, 21 and 60 (NK-TimeCourse). To determine the impact of infection on NK cells, splenocytes were isolated from spleens of naive or MCMV-infected mice at day 21 p.i. (NK-KLRG1). Following isolation, splenocytes were labeled with anti-CD45, anti-CD3, anti-CD19, anti-NK1.1, and anti-DX5 antibodies, and NK cells defined as CD45+, CD3-, CD19-, NK1.1+, and DX5+ cells, were separated from the mixture using fluorescence-activated cell sorting on FACSAriaIIu using a 70-µm nozzle. Sort purity was determined by sorting an aliquot of cells into 10% RPMI and then immediately reanalyzing the sorted aliquot by flow cytometry. In general, we achieved sort purities of > 98%. NK cells for RNA isolation were sorted directly into RLT lysis buffer (Qiagen) and their total RNA was isolated using RNeasy Micro Kit (Qiagen), according to the manufacturer's recommendations. The quality and quantity of isolated total RNA were then estimated on Agilent Bioanalyzer 2100 using the Agilent RNA 6000 Nano Kit, and only samples with RNA integrity number values higher than 9.0 were used. Before library generation, RNA was subjected to DNase I digestion (Thermo Fisher Scientific) followed by RNeasy MinElute column clean up (Qiagen). RNA-seq libraries were generated using the SMARTSeq v4 Ultra Low Input RNA Kit (Clontech Laboratories) as per the manufacturer's recommendations. From cDNA, final libraries were generated using the Nextera XT DNA Library Preparation Kit (Illumina). Concentrations of the final libraries were measured with a Qubit 2.0 Fluorometer (Thermo Fisher Scientific), and fragment length distribution was analyzed with the DNA High Sensitivity Chip on an Agilent 2100 Bioanalyzer (Agilent Technologies). All samples were normalized to 2 nM and pooled at equimolar

concentrations. The library pool was sequenced on the NextSeq500 (Illumina) in a single 1 × 76–bp run, producing 20.0–23.8 M reads per sample from a total of twelve mRNA-seq libraries for the NK-KLRG1 RNASeq experiment. For the NK-TimeCourse RNASeq experiment, the library pool was sequenced on the NextSeq500 (Illumina) in a single 1 x 72-bp run, producing 15.6-24.0 M reads per sample from 16 mRNA-Seq libraries. For both experiments and before downstream data processing and analysis, adapter sequences were hard-clipped from raw sequencing reads as part of the bcl2fastq pipeline (version 2.20.0.422).

Computing environment. Downstream RNA-Seq data processing and analysis were performed on a workstation equipped with an AMD Ryzen 5950X, 64 GB of RAM, and running Ubuntu 22.04.2 LTS (www.ubuntu.com), with the latest updates applied to the OS. Identical versions of the operating system, tools for RNASeq analysis, and DNA sequences have been used to analyze both NK-KLRG1 and NK-TimeCourse RNASeq experiments, and data analysis was facilitated by materials published by the Harvard Chan Bioinformatics Core [1].

Mouse genome and transcriptome nucleotide sequences. The file containing the nucleotide sequence of the mouse GRCm39 primary genome assembly (GRCm39.primary_assembly.genome.fa.gz, release M32), the file containing nucleotide sequences of all mouse transcripts on the reference chromosomes (gencode.vM32.transcripts.fa.gz), and the file containing comprehensive gene annotation on the mouse primary assembly sequence regions (gencode.vM32.primary_assembly.annotation.gtf.gz) were downloaded from the GENCODE project [2] FTP site (*ftp.ebi.ac.uk/pub/databases/gencode/Gencode_mouse/release_M32/*).

Additional nucleotide sequences used to evaluate the composition of sequencing libraries

Additional sequence files have been retrieved from the following sources to assess library compositions before alignment/mapping: a file containing the 43rd (GRCh38.p13) release of the *H. sapiens* genome (GRCh38.primary_assembly.genome.fa.gz) was downloaded from the GENCODE project (Frankish et al. 2023) FTP site (*ftp.ebi.ac.uk/pub/databases/gencode/Gencode_human/release_43/*); files containing the sequence of the *S. cerevisiae* (GCF_000146045.2_R64_genomic.fna.gz), *E. coli* (GCF_000005845.2_ASM584v2_genomic.fna.gz), MCMV (GCA_000859805.1_ViralProj15181_genomic.fna.gz) and HCMV (GCA_027926695.1_TB40_E_-_HFF_genomic.fna.gz) genomes, as well as the file containing sequences of various adapters, primers, linkers and vectors (UniVec database), have been retrieved from the NCBI FTP directory at *ftp.ncbi.nlm.nih.gov* [3]. All nucleotide sequences were downloaded from their respective repositories on June 29th, 2023.

Quality control of the sequencing libraries. As a first step in pre-mapping QC, the quality of the trimmed sequencing reads obtained from the sequencing facility was evaluated with FastQC v0.12.1 [4]. Next, the composition of the sequencing libraries was assessed using FastQ Screen v0.15.3 [5] and bowtie2 v.2.5.1 [6] to align the sequencing reads to a custom database containing the mouse genome and additional nucleotide sequences described above that might be present as technical or biological contaminants in mouse RNA-Seq libraries. Lastly, sequencing reads in each library were aligned to the mouse genome sequence using STAR v2.7.10.b [7-9]. Each obtained alignment file was then indexed with samtools v1.17 [10], and additional RNA-seq-related quality metrics for each sorted alignment file were evaluated using QoRTs v1.3.6 [11]. Quality control data from individual analyses were then aggregated using MultiQC v1.14 [12], and the combined QC results indicated an absence of apparent biases or errors in the sequencing libraries and confirmed that their composition is consistent with the experimental design.

Gene expression estimation. After performing quality control, transcript-level gene expression estimates for all samples were obtained using salmon v1.10.0 [13] in a mapping-based mode.

Before quantifying RNA-Seq samples, a decoy-aware mouse transcriptome file and a mapping-based index were constructed according to salmon documentation available at https://salmon.readthedocs.io/en/latest/index.html.

Differential expression analysis. Differentially expressed genes were identified using R v4.3.1 [14], RStudio v2023.06.0, build 421 [15] and several R packages. Mouse transcript IDs were first associated with the corresponding Gene IDs using functionalities available within the *R Base Package* v4.3.1 [14], *GenomicFeatures* v1.52.1 [16], and *AnnotationDbi* v1.62.1 [17] R packages. Transcript-level expression estimates obtained with *salmon* v1.10.0 were then imported into R and summarized to gene-level expression estimates using the *tximport* v1.28.0 [18]. Imported expression estimates, together with the experimental metadata, were then used to create a DESeqDataSet data structure using the DESeqDataSetFromTximport function in the *DESeq2* v1.40.2 package [19]. Next, a pre-filtering step was applied to DESeqDataSet to remove genes that, across all samples for a given RNASeq experiment, have less than 10 reads assigned to them. The pre-filtered count data were then normalized, rlog-transformed, and the overall similarity between all samples was evaluated by principal component analysis (PCA), as implemented in *PCAtools* v2.12.0 [20]. Following PCA, the DESeq2 differential expression pipeline was invoked by applying the DESeq function to the initially created DESeqDataSet object, and results tables containing $\log_2$ fold changes and p_{adj} values for each contrast were then constructed by calling the DESeq2 results function, setting $p_{adj} < 0.01$ as a cut-off for statistical significance. Gene IDs in the results tables were then associated with the corresponding gene symbols and functional information using *biomaRt* v2.56.1 [21,22], and combined gene information and statistics were then merged with the count data from the DESeqDataSet object to facilitate further data visualization and interpretation.

Gene ontology over-representation analysis (GO-ORA). GO-ORA was performed with *clusterprofiler* v4.8.1 [23,24]. In particular, enrichment analysis for the 'biological process' subontology [25,26] was carried out to determine whether the list of differentially expressed genes between KLRG1$^+$ NK cells from MCMV-infected mice and KLRG1$^-$ NK cells from mock-infected mice is enriched in specific GO terms by invoking the enrichGO function of the *clusterprofiler* package, whereby the list of differentially expressed genes ($p_{adj} < 0.01$) was set as a testing dataset, and the list of genes obtained after the pre-filtering step was set as a background dataset. The same function was also used to establish whether the subclusters of differentially expressed genes between NK cells from 60-day-old and 7-day-old mice, that have similar expression patterns (co-expression subclusters), are enriched in particular GO terms. For this analysis, the list of genes in each co-expression subcluster was used as a testing dataset, and the list of differentially expressed genes between NK cells from 60-day-old and 7-day-old mice was used as a background dataset. In both cases, ont ='BP', pAdjustMethod = 'BH', and pvalueCutoff = 0.05 were set as additional arguments for the analysis.

RNA-Seq results visualization. Results of the PCA were visualized using the biplot function available in *PCAtools*. Heatmaps showing the expression of selected gene sets (transcription factors, effector molecules, etc.) were drawn by supplying normalized, rlog-transformed, and row-scaled count data to *ComplexHeatmap* v2.16.0 [27,28]. Differentially expressed genes between NK cells from 60-day-old and 7-day-old mice were grouped into clusters with similar expression patterns using k-means clustering and visualized using the Heatmap function of the *ComplexHeatmap* package. The optimal number of clusters for the DE genes between NK cells from 60-day-old and 7-day-old mice was chosen using the *NbClust* v3.0.1 [29]. This chosen number was subsequently supplied as an argument for the Heatmap function of the *ComplexHeatmap* package, along with the default settings for calculating the distance matrix (euclidean) and clustering method (complete). GO-ORA results were visualized using *ggplot2*

v3.4.2 [30]. Data wrangling and additional customizations of plots have been made within the R environment using the functionalities available in *R Base Package* v4.3.1 [14], *tidyverse* v2.0.0 [30], *extrafont* v.0.19 [31], *ggtext* v0.1.2 [32], *ggrepel* v0.9.3 [33], *cowplot* v1.1.1 [34] and *RcolorBrewer* v1.1-3 [35] packages.

Statistical analysis

Wald test, as implemented in the *DESeq2* R package [19], was used to identify differentially expressed genes between experimental conditions. The obtained p-values were adjusted for multiple testing using the Benjamini and Hochberg method (BH) built into the *DESeq2* package, and genes with adjusted p-values (p_{adj}) smaller than 0.01 were considered statistically significant. For GO-ORA, a hypergeometric test implemented in the *clusterProfiler* package [23,24] was used to identify overrepresented GO terms in the list of differentially expressed genes and GO terms with BH-adjusted p-values smaller than 0.05 were considered statistically significant.

Supplementary references:

1 Piper, M. E., Mistry, M., Liu, J., Gammerdinger, W. J. & Khetani, R. S. *hbctraining/Intro-to-rnaseq-hpc-salmon-flipped: Introduction to RNA-se q using Salmon Lessons from HCBC (first release)*. (Zenodo, 2022).
2 Frankish, A. *et al.* GENCODE: reference annotation for the human and mouse genomes in 2023. *Nucleic Acids Res* **51**, D942-D949, doi:10.1093/nar/gkac1071 (2023).
3 Sayers, E. W. *et al.* Database resources of the national center for biotechnology information. *Nucleic Acids Res* **50**, D20-D26, doi:10.1093/nar/gkab1112 (2022).
4 Andrews, S. *FastQC: a quality control tool for high throughput sequence data*, <http://www.bioinformatics.babraham.ac.uk/projects/fastqc> (2010).
5 Wingett, S. W. & Andrews, S. FastQ Screen: A tool for multi-genome mapping and quality control. *F1000Res* **7**, 1338, doi:10.12688/f1000research.15931.2 (2018).
6 Langmead, B. & Salzberg, S. L. Fast gapped-read alignment with Bowtie 2. *Nat Methods* **9**, 357-359, doi:10.1038/nmeth.1923 (2012).
7 Dobin, A. *et al.* STAR: ultrafast universal RNA-seq aligner. *Bioinformatics* **29**, 15-21, doi:10.1093/bioinformatics/bts635 (2013).
8 Dobin, A. & Gingeras, T. R. Mapping RNA-seq Reads with STAR. *Curr Protoc Bioinformatics* **51**, 11 14 11-11 14 19, doi:10.1002/0471250953.bi1114s51 (2015).
9 Dobin, A. & Gingeras, T. R. Optimizing RNA-Seq Mapping with STAR. *Methods Mol Biol* **1415**, 245-262, doi:10.1007/978-1-4939-3572-7_13 (2016).
10 Li, H. *et al.* The Sequence Alignment/Map format and SAMtools. *Bioinformatics* **25**, 2078-2079, doi:10.1093/bioinformatics/btp352 (2009).
11 Hartley, S. W. & Mullikin, J. C. QoRTs: a comprehensive toolset for quality control and data processing of RNA-Seq experiments. *BMC Bioinformatics* **16**, 224, doi:10.1186/s12859-015-0670-5 (2015).
12 Ewels, P., Magnusson, M., Lundin, S. & Kaller, M. MultiQC: summarize analysis results for multiple tools and samples in a single report. *Bioinformatics* **32**, 3047-3048, doi:10.1093/bioinformatics/btw354 (2016).
13 Patro, R., Duggal, G., Love, M. I., Irizarry, R. A. & Kingsford, C. Salmon provides fast and bias-aware quantification of transcript expression. *Nat Methods* **14**, 417-419, doi:10.1038/nmeth.4197 (2017).
14 R Core Team. *R: A Language and Environment for Statistical Computing*, <https://www.R-project.org/> (2023).
15 R. Studio Team. RStudio: Integrated Development Environment for R. (2019).

16 Lawrence, M. *et al.* Software for computing and annotating genomic ranges. *PLoS Comput Biol* **9**, e1003118, doi:10.1371/journal.pcbi.1003118 (2013).
17 Pagès, H., Carlson, M., Falcon, S. & Li, N. AnnotationDbi: Manipulation of SQLite-based annotations in Bioconductor. (2022).
18 Soneson, C., Love, M. I. & Robinson, M. D. Differential analyses for RNA-seq: transcript-level estimates improve gene-level inferences. *F1000Research* **4**, doi:10.12688/f1000research.7563.1 (2015).
19 Love, M. I., Huber, W. & Anders, S. Moderated estimation of fold change and dispersion for RNA-seq data with DESeq2. *Genome Biol* **15**, 550, doi:10.1186/s13059-014-0550-8 (2014).
20 Blighe, K. & Lun, A. PCAtools: PCAtools: Everything Principal Components Analysis. (2022).
21 Durinck, S. *et al.* BioMart and Bioconductor: a powerful link between biological databases and microarray data analysis. *Bioinformatics* **21**, 3439-3440, doi:10.1093/bioinformatics/bti525 (2005).
22 Durinck, S., Spellman, P. T., Birney, E. & Huber, W. Mapping identifiers for the integration of genomic datasets with the R/Bioconductor package biomaRt. *Nat Protoc* **4**, 1184-1191, doi:10.1038/nprot.2009.97 (2009).
23 Wu, T. Z. *et al.* clusterProfiler 4.0: A universal enrichment tool for interpreting omics data. *Innovation-Amsterdam* **2**, doi:ARTN 100141

10.1016/j.xinn.2021.100141 (2021).
24 Yu, G. C., Wang, L. G., Han, Y. Y. & He, Q. Y. clusterProfiler: an R Package for Comparing Biological Themes Among Gene Clusters. *Omics* **16**, 284-287, doi:10.1089/omi.2011.0118 (2012).
25 Ashburner, M. *et al.* Gene ontology: tool for the unification of biology. The Gene Ontology Consortium. *Nat Genet* **25**, 25-29, doi:10.1038/75556 (2000).
26 Gene Ontology Consortium. The Gene Ontology resource: enriching a GOld mine. *Nucleic Acids Res* **49**, D325-D334, doi:10.1093/nar/gkaa1113 (2021).
27 Gu, Z. Complex Heatmap Visualization. *iMeta*, doi:10.1002/imt2.43 (2022).
28 Gu, Z., Eils, R. & Schlesner, M. Complex heatmaps reveal patterns and correlations in multidimensional genomic data. *Bioinformatics*, doi:10.1093/bioinformatics/btw313 (2016).
29 Charrad, M., Ghazzali, N., Boiteau, V. & Niknafs, A. NbClust: An R Package for Determining the Relevant Number of Clusters in a Data Set. *Journal of Statistical Software* **61**, 1 - 36, doi:10.18637/jss.v061.i06 (2014).
30 Wickham, H. *et al.* Welcome to the Tidyverse. *Journal of Open Source Software* **4**, 1686, doi:10.21105/joss.01686 (2019).
31 Chang, W. extrafont: Tools for Using Fonts. (2023).
32 Wilke, C. O. & Wiernik, B. M. ggtext: Improved Text Rendering Support for 'ggplot2'. (2022).
33 Slowikowski, K. ggrepel: Automatically Position Non-Overlapping Text Labels with 'ggplot2'. (2022).
34 Wilke, C. O. cowplot: Streamlined Plot Theme and Plot Annotations for 'ggplot2'. (2020).
35 Neuwirth, E. RColorBrewer: ColorBrewer Palettes. (2022).
