# Supplementary material for: Perinatal murine cytomegalovirus infection reshapes the transcriptional profile and functionality of NK cells
Source: Nat Commun. 2023 Oct 12;14:6412. doi: 10.1038/s41467-023-42182-w (PMC10570381; doi:10.1038/s41467-023-42182-w)
Supplement: Supplementary file 3 — Reporting Summary [file 41467_2023_42182_MOESM3_ESM.pdf]

nature portfolio

Corresponding author(s): Stipan Jonjić & Ilija Brizić

Last updated by author(s): Jul 18, 2023

Reporting Summary

Nature Portfolio wishes to improve the reproducibility of the work that we publish. This form provides structure for consistency and transparency in reporting. For further information on Nature Portfolio policies, see our Editorial Policies and the Editorial Policy Checklist.

Statistics

For all statistical analyses, confirm that the following items are present in the figure legend, table legend, main text, or Methods section.

n/a	Confirmed	
☐	☒	The exact sample size (n) for each experimental group/condition, given as a discrete number and unit of measurement
☐	☒	A statement on whether measurements were taken from distinct samples or whether the same sample was measured repeatedly
☐	☒	The statistical test(s) used AND whether they are one- or two-sided<br>*Only common tests should be described solely by name; describe more complex techniques in the Methods section.*
☐	☒	A description of all covariates tested
☐	☒	A description of any assumptions or corrections, such as tests of normality and adjustment for multiple comparisons
☐	☒	A full description of the statistical parameters including central tendency (e.g. means) or other basic estimates (e.g. regression coefficient) AND variation (e.g. standard deviation) or associated estimates of uncertainty (e.g. confidence intervals)
☐	☒	For null hypothesis testing, the test statistic (e.g. F, t, r) with confidence intervals, effect sizes, degrees of freedom and P value noted<br>*Give P values as exact values whenever suitable.*
☒	☐	For Bayesian analysis, information on the choice of priors and Markov chain Monte Carlo settings
☒	☐	For hierarchical and complex designs, identification of the appropriate level for tests and full reporting of outcomes
☒	☐	Estimates of effect sizes (e.g. Cohen's d, Pearson's r), indicating how they were calculated

Our web collection on statistics for biologists contains articles on many of the points above.

Software and code

Policy information about availability of computer code

Data collection	Flow cytometry data were collected using BD FACSDiva (v8.02). RT-PCR data were collected using 7500 Fast Real Time PCR machine (ABI).
Data analysis	GraphPad Prism (v8), FlowJo (v10), STAR v2.7.10b, bowtie2 v2.5.1, salmon v1.10.0, FastQC v0.12.1, multiqc v1.14, FastQ Screen v0.15.3, samtools v1.17, QoRTs v1.3.6, R v4.3.1. Attached R packages: org.Mm.eg.db_3.17.0, clusterProfiler_4.8.1, S4Vectors_0.38.1, ComplexHeatmap_2.16.0, RColorBrewer_1.1-3, biomaRt_2.56.1, cowplot_1.1.1, ggtext_0.1.2, extrafont_0.19, PCAtools_2.12.0, ggrepel_0.9.3, ggplot2_3.4.2, DESeq2_1.40.2, SummarizedExperiment_1.30.2, MatrixGenerics_1.12.2, matrixStats_1.0.0, stringr_1.5.0, tximport_1.28.0, GenomicFeatures_1.52.1, AnnotationDbi_1.62.1, Biobase_2.60.0, GenomicRanges_1.52.0, GenomeInfoDb_1.36.1, IRanges_2.34.1, BiocGenerics_0.46.0.

For manuscripts utilizing custom algorithms or software that are central to the research but not yet described in published literature, software must be made available to editors and reviewers. We strongly encourage code deposition in a community repository (e.g. GitHub). See the Nature Portfolio guidelines for submitting code & software for further information.

Data

Policy information about availability of data

All manuscripts must include a data availability statement. This statement should provide the following information, where applicable:

- Accession codes, unique identifiers, or web links for publicly available datasets
- A description of any restrictions on data availability
- For clinical datasets or third party data, please ensure that the statement adheres to our policy

The RNA sequencing data data generated in this study have been deposited in the European nucleotide archive under accession code PRJEB64583. Datasets generated during and/or analyzed in this study are available from the corresponding author upon request. Source data are provided with this paper.

Research involving human participants, their data, or biological material

Policy information about studies with human participants or human data. See also policy information about sex, gender (identity/presentation), and sexual orientation and race, ethnicity and racism.

Reporting on sex and gender	N/A
Reporting on race, ethnicity, or other socially relevant groupings	N/A
Population characteristics	N/A
Recruitment	N/A
Ethics oversight	N/A

Note that full information on the approval of the study protocol must also be provided in the manuscript.

Field-specific reporting

Please select the one below that is the best fit for your research. If you are not sure, read the appropriate sections before making your selection.

- [x] Life sciences
- [ ] Behavioural & social sciences
- [ ] Ecological, evolutionary & environmental sciences

For a reference copy of the document with all sections, see nature.com/documents/nr-reporting-summary-flat.pdf

Life sciences study design

All studies must disclose on these points even when the disclosure is negative.

Sample size	Experimental sample sizes were selected according to commonly accepted standards for analysis of immune response in mouse models to achieve statistical significance. Group sizes for in vitro experiments were selected on the basis of prior knowledge of variation.<br><br>No statistical method was used to predetermine sample size. Sample size was chosen based on our previous studies using the same types of assays, as well as published literature. Sample sizes were determined based on the current standard, as the minimum amount of mice required to detect significance with an alpha rate set at .05 in a standardly powered experiment.
Data exclusions	We did not exclude animals or data from the study.
Replication	All experiments were replicated. Number of reproductions of each experimental finding is stated in figure legends.
Randomization	Newborn mice were randomized in each experiment.
Blinding	Blinding was not done, as the knowledge of grouping was essential to perform these studies. In addition, blinding in this type of studies is not required.

Reporting for specific materials, systems and methods

We require information from authors about some types of materials, experimental systems and methods used in many studies. Here, indicate whether each material, system or method listed is relevant to your study. If you are not sure if a list item applies to your research, read the appropriate section before selecting a response.

Materials & experimental systems

n/a	Involved in the study
	☒ Antibodies
	☒ Eukaryotic cell lines
☒	☐ Palaeontology and archaeology
	☒ Animals and other organisms
☒	☐ Clinical data
☒	☐ Dual use research of concern
☒	☐ Plants

Methods

n/a	Involved in the study
☒	☐ ChIP-seq
	☒ Flow cytometry
☒	☐ MRI-based neuroimaging

Antibodies

Antibodies used

For flow cytometry:
The following antibodies were purchased from ThermoFisher:
anti-mouse CD45.2 (clone 104) ef506 # 69-0454-82 (dilution 1:300)
anti-mouse CD279/PD-1 (clone J43) APC # 17-9985-82 (dilution 1:100)
anti-mouse CD62L (clone MEL-14) PE-Cy7 # 25-0621-82 (dilution 1:400)
anti-mouse CD69 (clone H1.2F3) FITC # 11-0691-82 (dilution 1:300)
anti-mouse CD103 (clone 2E7) PE # 12-1031-82 (dilution 1:200)
anti-mouse IFN-γ (clone XMG1.2) FITC # 17-7311-82 (dilution 1:100)
anti-mouse Bcl-2 (clone 10C4) FITC # 11-6992-42 (dilution 1:100)
anti-mouse/human T-bet (clone 4B10) PE-Cy7 # 25-5825-82 (dilution 1:100)
anti-mouse CD25 (clone PC61.5) PE-Cy7 # 25-0251-82 (dilution 1:400)
anti-mouse CD3ε (clone 145-2C11) PerCPCy5.5 #45-0031-82 (dilution 1:100)
anti-mouse CD19 (clone eBio1D3) PerCPCy5.5 #45-0193-82 (dilution 1:400)
anti-mouse CD49b (clone DX5) FITC # 11-5971-82 (dilution 1:100)
anti-mouse NK1.1 (clone PK136) APC # 17-5941-82 (dilution 1:100)
anti-mouse NK1.1 (clone PK136) PE-eFluor 610 #61-5941-82 (dilution 1:100)
anti-mouse CD127 (clone SB/199) PE # 12-1273-82 (dilution 1:100)
anti-mouse CD335 (NKp46) (clone 29A1.4) FITC #11-3351-82 (dilution 1:100)
anti-mouse CD335 (NKp46) (clone 29A1.4) PE-eFluor 610 # 61-3351-82 (dilution 1:100)
anti-mouse CD335 (NKp46) (clone 29A1.4) PE # 12-3351-82 (dilution 1:100)
anti-mouse CD27 (clone LG.7F9) PE-Cy7 # 25-0271-82 (dilution 1:100)
anti-mouse CD11b (clone M1/70) FITC # 11-0112-82 (dilution 1:400)
anti-mouse CD122 (clone TM-b1) FITC # 12-1222-82 (dilution 1:100)
anti-mouse Ly-49H (clone 3D10) APC # 17-5886-82 (dilution 1:200)
anti-mouse KLRG1 (clone 2F1) PE-eFluor 610 # 61-5893-82 (dilution 1:100)
anti-mouse NKG2D (clone C7) PE-Cy7 # 25-5882-82 (dilution 1:100)
anti-mouse CD94 (clone 18d3) PE # 12-0941-81 (dilution 1:100)
anti-mouse Ly-49G2 (clone 4D11) FITC # 11-5781-82
anti-mouse Ly-49I (clone YLI-90) PE # A15414 (dilution 1:100)
anti-mouse Ly-49A/D (clone eBio12A8 (12A8)) PE # 12-5783-81 (dilution 1:100)
anti-mouse Eomes (clone Dan11Mag) PE # 12-4875-82 (dilution 1:100)
anti-mouse CD200R (clone OX-110) PE # MA1-82713 (dilution 1:100)
anti-mouse CD244.1 (2B4) (clone C9.1) PE # 17-2440-82 (dilution 1:100)
anti-mouse Ly6c (clone HK1.4) APC-eFluor780 # 47-5932-82 (dilution 1:100)
anti-mouse CD183 (CXCR3) (clone CXCR3-173) APC # 17-1831-82 (dilution 1:100)
anti-mouse CD218a (IL-18Ra) (clone P3TUNYA) PE # 12-5183-82 (dilution 1:100)
anti-mouse CD226 (DNAM-1) (clone 10E5) APC # 17-2261-82 (dilution 1:400)
anti-mouse NKG2A/C/E (clone 20D5) PerCP-eFluor710 # 46-5896-82 (dilution 1:100)
anti-mouse CD336 (Tim3) (clone RMT3-23) PE-Cy7 # 25-5870-82 (dilution 1:100)
anti-mouse MHC-I (clone 28-14-8) APC # 17-5999-82 (dilution 1:100)
anti-mouse CD223 (Lag-3) (clone C9B7W) eFluor450 # 48-2231-82 (dilution 1:100)
anti-mouse TIGIT (clone GIGD7) PE-Cy7 # 48-2231-82 (dilution 1:100)
anti-mouse TER-119 (clone TER-119) PE # 25-5921-82 (dilution 1:100)
anti-mouse CD45R (B220) (clone RA3-6B2) FITC # 11-0452-82 (dilution 1:100)
anti-mouse Ki67 (clone SolA15) PerCP-eFluor710 # 46-5698-82 (dilution 1:100)
anti-mouse BLIMP-1 (clone 5E7) PE # 12-9850-82 (dilution 1:100)
he following antibody were purchased from BioLegend:
anti-mouse CD49a (clone HMα1) APC #142605 (dilution 1:100)
The following antibody were purchased from Miltenyi Biotec:
anti-mouse Ly49A (REA1018) VioBlue 130-117-113F (dilution 1:50)
The following antibodies were purchased from Cell Signaling:
anti-mouse TCF-1 (clone C6309) PE-Cy7 #90511S (dilution 1:400)

anti-mouse TCF-1 (clone C6309) af488 #6444S (dilution 1:400)
For depletion experiments antiboies were purchased from BioXCell:
anti-NK1.1 (clone PK136, #BE0036), anti-CD4 (clone GK1.5, #BE0003-1) and anti-CD8 antibody (clone 2.43, #BE0061)

Validation: All antibodies in this study are commercially available and have been validated by manufacturer. Any validation statements are available on the manufacturer's website.

Eukaryotic cell lines

Policy information about cell lines and Sex and Gender in Research

Cell line source(s)	RMA-S cell line was provided by Wayne M. Yokoyama.
Authentication	Cell line was not authenticated.
Mycoplasma contamination	Cell line was not tested for mycoplasma contamination.
Commonly misidentified lines (See ICLAC register)	None

Animals and other research organisms

Policy information about studies involving animals; ARRIVE guidelines recommended for reporting animal research, and Sex and Gender in Research

Laboratory animals	Laboratory mice ranging in age from one day to four months old were utilized in the study. The following strains were used: C57BL/6J, BALB/c, 129/SvJ, Rag2-/-γc-/-, IL12-/-, Ifnar1-/-, Klrk-/-, Ncr1iCre Eomesf/f, Tgfbr2f/fNcr1iCre.
Wild animals	The study did not involve wild animals.
Reporting on sex	In the study we used newborn mice of both genders and both genders were included in the data presented in the manuscript. Data on gender for each experiment was not collected, as we did not observe apparent variations in individual results for major phenotypes.
Field-collected samples	The study did not involve samples collected from the field.
Ethics oversight	The National Ethics Committee for the Protection of Animals Used for Scientific Purposes (Ministry of Agriculture) approved animal experiments (UP/I-322-01/18-01/30).

Note that full information on the approval of the study protocol must also be provided in the manuscript.

Flow Cytometry

Plots

Confirm that:

- [x] The axis labels state the marker and fluorochrome used (e.g. CD4-FITC).
- [x] The axis scales are clearly visible. Include numbers along axes only for bottom left plot of group (a 'group' is an analysis of identical markers).
- [x] All plots are contour plots with outliers or pseudocolor plots.
- [x] A numerical value for number of cells or percentage (with statistics) is provided.

Methodology

Sample preparation	Single-cell suspensions of spleen, liver, lung, bone marrow, and blood leukocytes were prepared using standard protocols. Before staining of lymphocytes, Fc receptors were blocked using a 2.4G2 antibody. Antibodies previously listed were used. Fixable Viability Dye (Thermo Fisher) was used to exclude dead cells. Intracellular staining, permeabilization, and fixation of cells were done using the Fixation/Permeabilization kit (Thermo Fisher Scientific).
Instrument	BD Aria III
Software	BD Facs Diva (v8.02) was used for data colection, FlowJo (v10.7.0) was used for data analysis
Cell population abundance	Sort purity was determined by sorting an aliquot of cells into 10% RPMI and then immediately reanalyzing the sorted aliquot by flow cytometry. In general, we achieved sort purities of > 98%.<br>Cellular size and viability stain (Fixable Viability Dye (ThermoFisher)) were used to exclude dead cells. Positive populations were defined using unstained cells as reference.

Gating strategy	All gates were set based on single-stained compensation controls.

☒ Tick this box to confirm that a figure exemplifying the gating strategy is provided in the Supplementary Information.
